# Isolation and characterization of microorganisms capable of cleaving the ether bond of 2-phenoxyacetophenone

**DOI:** 10.1038/s41598-022-06816-1

**Published:** 2022-02-21

**Authors:** Saki Oya, Satoshi Tonegawa, Hirari Nakagawa, Hiroshi Habe, Toshiki Furuya

**Affiliations:** 1 Department of Applied Biological Science, Faculty of Science and Technology, Tokyo University of Science, 2641 Yamazaki, Noda, Chiba 278-8510 Japan; 2 Environmental Management Research Institute, National Institute of Advanced Industrial Science and Technology (AIST), 16-1 Onogawa, Tsukuba, Ibaraki 305-8569 Japan

**Keywords:** Biotechnology, Microbiology

## Abstract

Lignin is a heterogeneous aromatic polymer and major component of plant cell walls. The β-*O*-4 alkyl aryl ether is the most abundant linkage within lignin. Given that lignin is effectively degraded on earth, as yet unknown ether bond–cleaving microorganisms could still exist in nature. In this study, we searched for microorganisms that transform 2-phenoxyacetophenone (2-PAP), a model compound for the β-*O*-4 linkage in lignin, by monitoring ether bond cleavage. We first isolated microorganisms that grew on medium including humic acid (soil-derived organic compound) as a carbon source. The isolated microorganisms were subsequently subjected to colorimetric assay for 2-PAP ether bond–cleaving activity; cells of the isolated strains were incubated with 2-PAP, and strains producing phenol via ether bond cleavage were selected using phenol-sensitive Gibbs reagent. This screening procedure enabled the isolation of various 2-PAP–transforming microorganisms, including 7 bacteria (genera: *Acinetobacter*, *Cupriavidus*, *Nocardioides*, or *Streptomyces*) and 1 fungus (genus: *Penicillium*). To our knowledge, these are the first microorganisms demonstrated to cleave the ether bond of 2-PAP. One Gram-negative bacterium, *Acinetobacter* sp. TUS-SO1, was characterized in detail. HPLC and GC–MS analyses revealed that strain TUS-SO1 oxidatively and selectively cleaves the ether bond of 2-PAP to produce phenol and benzoate. These results indicate that the transformation mechanism differs from that involved in reductive β-etherase, which has been well studied. Furthermore, strain TUS-SO1 efficiently transformed 2-PAP; glucose-grown TUS-SO1 cells converted 1 mM 2-PAP within only 12 h. These microorganisms might play important roles in the degradation of lignin-related compounds in nature.

## Introduction

Lignin is a heterogeneous aromatic polymer that forms plant cell walls, together with cellulose and hemicellulose. Lignin is the second most-abundant organic substance on earth, next to cellulose. Lignin is composed of *p*-hydroxyphenyl, guaiacyl, and syringyl units linked by ether and carbon–carbon bonds. Based on its abundance and composition, lignin has attracted attention as a renewable organic substance derived from plant biomass^1,2^. In particular, lignin is potentially useful for the production of industrial chemicals, such as aromatic compounds, that are currently produced from petroleum. Microorganisms effectively degrade lignin in nature and play important roles in the global carbon cycle. Elucidating how microorganisms degrade lignin would be helpful to not only enhance understanding of the carbon cycle but could also facilitate the biotechnological application of these organisms to lignin valorization^3–5^.

Cleavage of ether bonds is an important step in microbial lignin degradation, as aromatic constituents are primarily linked via strong, covalent ether bonds. Degradation of lignin in nature is generally initiated by extracellular enzymes produced by fungi^6–8^. For example, white-rot fungi produce extracellular peroxidases and laccases, which abstract electrons from lignin either directly or via mediators. The resultant positively charged radical cations undergo a number of reactions that lead to the formation of a variety of products, including compounds generated after cleavage of the ether bonds. Recently, it was also reported that several types of bacteria produce extracellular peroxidases and laccases that degrade lignin^3,9^. In addition, Sphingomonad bacteria can degrade low-molecular-weight lignin-related compounds using intracellular enzymes^4,5,10–12^. For example, guaiacylglycerol-β-guaiacyl ether is converted to α-(2-methoxyphenoxy)-β-hydroxypropiovanillone (MPHPV) through the oxidation of the α-carbon. The resulting MPHPV is converted to α-glutathionyl-β-hydroxypropiovanillone (GS-HPV) and guaiacol through the nucleophilic attack of glutathione on the MPHPV β-carbon atom. This ether bond cleavage is catalyzed by glutathione *S*-transferases, which are also known as β-etherases. Finally, GS-HPV is converted to β-hydroxypropiovanillone and oxidized glutathione^13,14^.

Given that lignin is effectively degraded by microbial consortia including white-rot fungi and Sphingomonad bacteria, it is possible that as yet unknown ether bond–cleaving fungi and bacteria exist in nature. The β-*O*-4 alkyl aryl ether is the most abundant linkage within lignin (40–60%, depending on lignin type)^4,5^. Lignin model compounds (LMCs) such as guaiacylglycerol-β-guaiacyl ether, veratrylglycerol-β-guaiacyl ether, and 2-phenoxyactophenone (2-PAP) are often used to study cleavage of the β-*O*-4 linkage^6,9,15,16^. With regard to 2-PAP, transformation of this compound via chemical methods has been well studied^15–18^. However, surprisingly, to date there have been no reports describing microorganisms that degrade or transform 2-PAP. Screening microorganisms for 2-PAP transformation capability could lead to the discovery of novel ether bond–cleaving fungi and/or bacteria.

Conventional methods to screen for LMC-degrading or transforming microorganisms are based on monitoring growth on LMCs as a carbon source^19,20^. However, these methods are not suitable for isolating microorganisms that can cleave LMC ether bonds but do not have downstream pathways to assimilate the LMCs. Microorganisms that can cleave LMC ether bonds but do not grow in the presence of these compounds due to their antimicrobial activity also cannot be isolated using such methods. In contrast, monitoring the formation of transformation products after incubation with an LMC as a substrate provides a direct screening method for microorganisms that can cleave LMC ether bonds. Synthetic compounds that yield fluorogenic products upon cleavage readily enable the detection of target organisms^21,22^. However, these compounds are not available commercially, and their synthesis tends to be laborious.

This study was aimed to isolate microorganisms that have new ether bond–cleaving enzymes. We screened for microorganisms that transform 2-PAP using a direct screening method based on detection of ether bond–cleaving activity. Microorganisms isolated from soils were incubated with 2-PAP. We hypothesized that phenol would be produced from 2-PAP as a result of microbial cleavage of the ether bond. Strains that cleaved 2-PAP were selected using a colorimetric assay with a commercially available reagent sensitive to phenol. Using this screening procedure, a variety of 2-PAP ether bond–cleaving microorganisms, including 7 bacteria and 1 fungus, were isolated. Herein, we report the results of studies characterizing these microorganisms and detailed investigation of the 2-PAP transformation pathway in the bacterial isolate that exhibited high 2-PAP-transforming activity.

## Results

### Screening for microorganisms that transform 2-PAP via ether bond cleavage

In the first screening, we examined microbial growth on humic acid as a carbon source. Humic acid is a fraction of soil organic matter derived primarily from plants and includes organic compounds with a wide molecular weight range, such as aromatic polymers^23^. A variety of microorganisms can utilize humic acid for growth^24,25^. In addition, genes involved in the degradation of aromatic compounds are reportedly up-regulated in the presence of humic acid^26^. Microorganisms from soil samples were cultivated for 1 or 2 weeks on basal medium supplemented with 1 g/L commercially available nitrohumic acid as a carbon source and 18 g/L agar. After cultivation of microorganisms from 540 soil samples, we isolated 1183 candidate strains that utilized nitrohumic acid.

**Figure 1:**
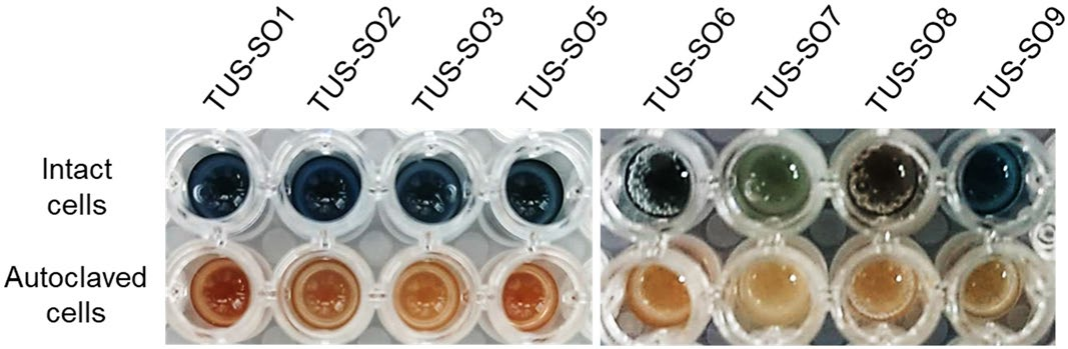
Colorimetric assay of 2-PAP ether bond–cleaving activity. Microbial cells after cultivation on solid basal medium supplemented with nitrohumic acid were suspended in liquid basal medium supplemented with 0.1 g/L yeast extract and 1% (v/v) Tween 80. The cell suspension was incubated with 5 mM 2-PAP for 72 h. The assay was performed by adding Gibbs reagent to the mixture following the reaction between intact cells of the isolated strains and 2-PAP. Control reactions were performed using autoclaved cells of the isolated strains.

**Figure 2:**
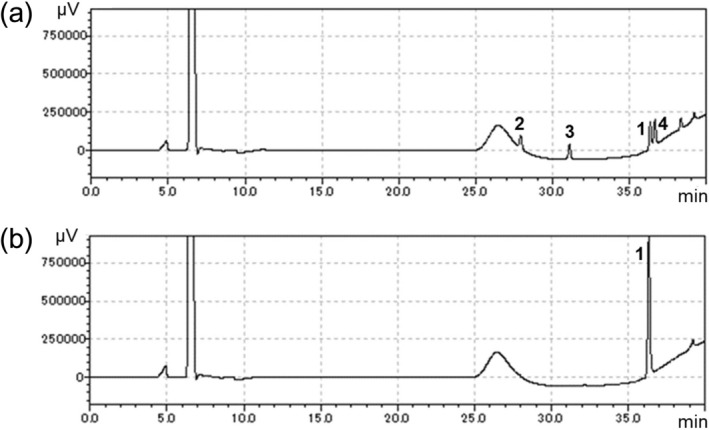
HPLC analysis of the reaction products formed by incubation of strain TUS-SO1 with 2-PAP. Microbial cells after cultivation on solid basal medium supplemented with nitrohumic acid were suspended in liquid basal medium supplemented with 0.1 g/L yeast extract and 1% (v/v) Tween 80. The cell suspension was incubated with 1 mM 2-PAP for 72 h. Intact cells (**a**) or autoclaved cells (**b**) of strain TUS-SO1 were incubated with 2-PAP. Peaks 1 (at 36.3 min), 2 (at 27.7 min), 3 (at 31.0 min), and 4 (at 36.6 min) correspond to 2-PAP, phenol, benzoate, and 2-PPE, respectively.

In the second screening, we assayed the isolated microorganisms for cleavage of the 2-PAP ether bond. Microbial cells cultivated on solid basal medium supplemented with nitrohumic acid were suspended in liquid basal medium supplemented with 0.1 g/L yeast extract and 1% (v/v) Tween 80 and incubated with 5 mM 2-PAP as a substrate for 72 h. Yeast extract was added as a potential cofactor and Tween 80 was added to increase permeability of microbial cell membranes to the substrate^27^. Strains producing phenol via ether bond cleavage of 2-PAP were selected using a colorimetric assay with phenol-sensitive Gibbs reagent^28,29^. A total of 8 strains from among the 1,183 isolates exhibited a positive color change (strains TUS-SO1, -SO2, -SO3, -SO5, -SO6, -SO7, -SO8, and -SO9) (Fig. 1). Incubation of autoclaved cells of these strains with 2-PAP resulted in no color change. The reaction products were further analyzed using high-performance liquid chromatography (HPLC), which revealed a marked decrease in the level of 2-PAP after incubation with the 8 positive strains (Figs. 2 and S1). In addition, a product with a retention time of 27.7 min coincided with an authentic sample of phenol after incubation of the positive strains with 2-PAP (Figs. 2, S1a-d,f,g, and S2), excluding TUS-SO7 (Fig. S1e). Strain TUS-SO7 generated two products with retention times of 32.9 min and 33.8 min that could be phenol derivatives. These results suggest that this method is useful for screening microorganisms for the ability to cleave the ether bond of 2-PAP.

### Taxonomic identification of microorganisms that transform 2-PAP

**Figure 3:**
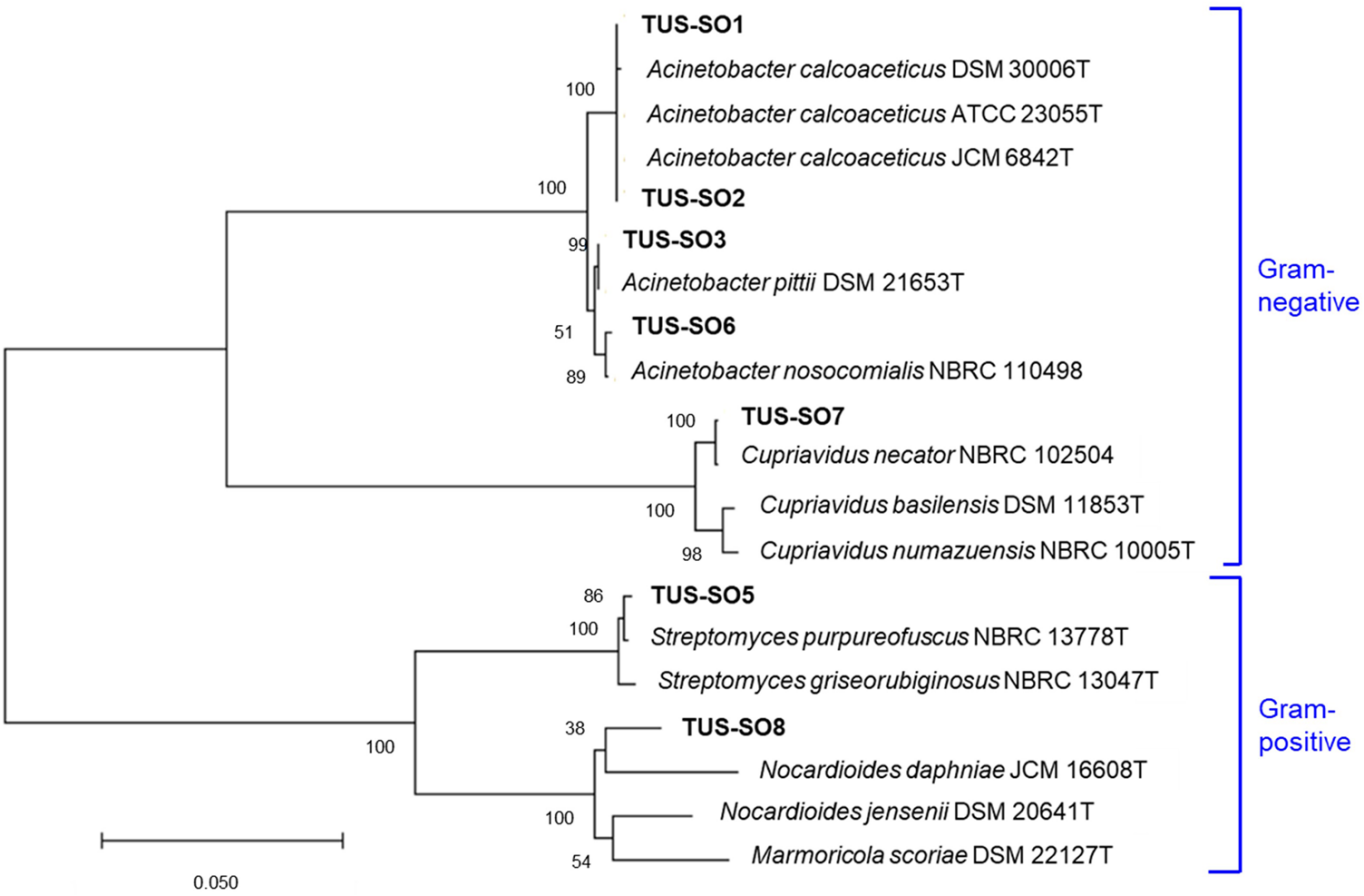
Phylogenetic relationships of 2-PAP–transforming bacteria isolated in this study based on the 16S rRNA gene sequence. Bootstrap values from 1000 replications are shown at each of the branch points on the tree.

The strains were taxonomically identified by sequencing the 16S rRNA gene or ITS1 region (Table 1). Seven of the isolates were classified as bacteria, and one isolate (strain TUS-SO9) was classified as a fungus of the genus *Penicillium*. A phylogenetic tree of the 16S rRNA gene sequences was constructed (Fig. 3). The bacteria belonged to 4 different genera, *Acinetobacter* (TUS-SO1, TUS-SO2, TUS-SO3, and TUS-SO6), *Streptomyces* (TUS-SO5), *Cupriavidus* (TUS-SO7), and *Nocardioides* (TUS-SO8). *Acinetobacter* and *Cupriavidus* are Gram-negative organisms of the phylum Proteobacteria, whereas *Streptomyces* and *Nocardioides* are Gram-positive organisms of the phylum Actinobacteria. 2-PAP–transforming microorganisms belonging to a variety of genera were obtained through the screening method used in this study. *Acinetobacter* sp. TUS-SO1 exhibited high and stable 2-PAP–transforming activity (Fig. S2) and was thus chosen for further analysis.

**Table 1:** 2-PAP–transforming microorganisms isolated in this study.

Strain	Class/Order	Accession no	Closest type strain (accession no.)	Similarity (%)
TUS-SO1	*Gammaproteobacteria*/*Pseudomonadales*	LC602842	*Acinetobacter calcoaceticus* ATCC 23055 T (NR_117619.1)	1448/1448 (100)
TUS-SO2	*Gammaproteobacteria*/*Pseudomonadales*	LC602843	*Acinetobacter calcoaceticus* ATCC 23055 T (NR_117619.1)	1422/1426 (99)
TUS-SO3	*Gammaproteobacteria*/*Pseudomonadales*	LC602844	*Acinetobacter pittii* DSM 21653 T (NR_117621.1)	1447/1448 (99)
TUS-SO5	*Actinobacteria*/*Streptomycetales*	LC602845	*Streptomyces purpureofuscus* NBRC 13778 T (AB184475.2)	1313/1318 (99)
TUS-SO6	*Gammaproteobacteria*/*Pseudomonadales*	LC602846	*Acinetobacter nosocomialis* NBRC 110498 (LC014141.1)	1442/1446 (99)
TUS-SO7	*Betaproteobacteria*/*Burkholderiales*	LC602847	*Cupriavidus necator* NBRC 102504 (AB681838.1)	1415/1424 (99)
TUS-SO8	*Actinobacteria*/*Propionibacteriales*	LC602848	*Nocardioides jensenii* DSM 20641 T (NR_119353.1)	1348/1394 (97)
TUS-SO9	*Eurotiomycetes/Eurotiales*	LC602849	*Penicillium citrinum* JCM 22607 (LC228681.1)	217/217 (100)

### Identification of products generated from 2-PAP transformation by TUS-SO1

**Figure 4:**
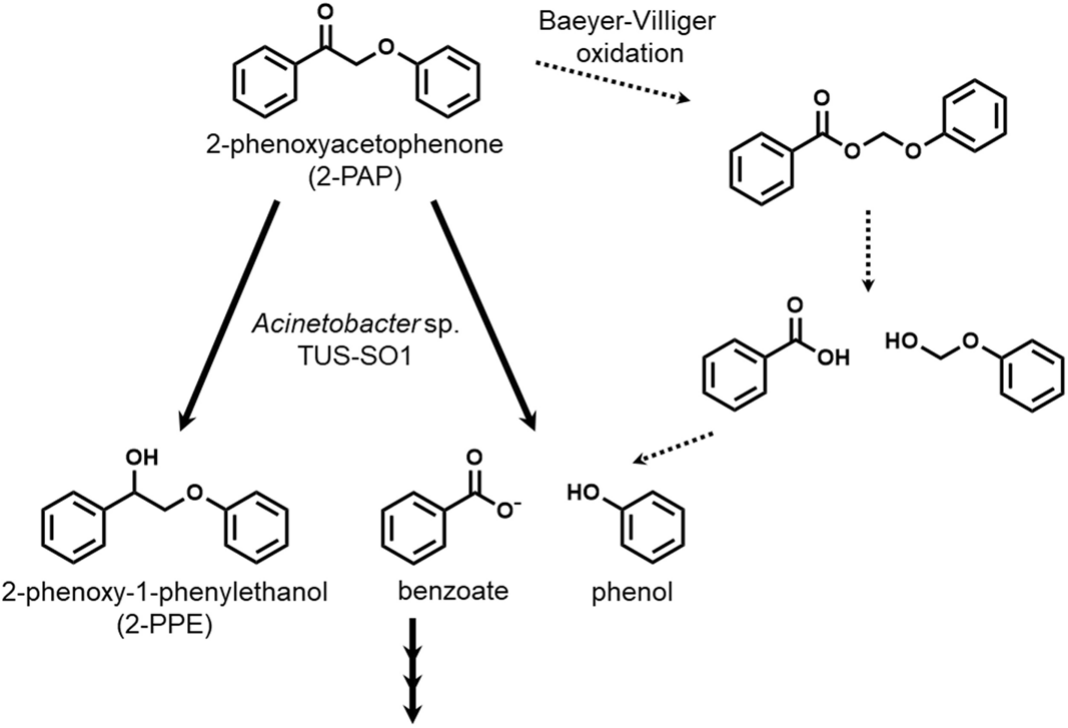
Proposed pathway of 2-PAP transformation by strain TUS-SO1. The dotted arrow indicates the reported chemical transformation pathway via Baeyer–Villiger oxidation.

We analyzed the products generated by incubation of 2-PAP with strain TUS-SO1 in detail. HPLC analysis of the reaction products revealed 3 peaks (retention times of 27.7 min, 31.0 min, and 36.6 min) in addition to the substrate peak (36.3 min) (Fig. 2). These products were not detected in the control reaction using autoclaved TUS-SO1 cells. Furthermore, the retention time and UV–visible absorption spectrum of each product were consistent with those of an authentic sample of phenol, benzoate, or 2-phenoxy-1-phenylethanol (2-PPE), respectively (Figs. 2 and S3). In addition, gas chromatography–mass spectrometry (GC–MS) analysis revealed products with parent ion *m/z* values of 94 and 214. The GC–MS spectra of the *m/z* 94 and 214 products were consistent with those of authentic samples of phenol and 2-PPE, respectively (Figs. S4 and S5). Furthermore, after treatment of the reaction mixture with trimethylsilyl derivatization reagent, the derivative of benzoate was detected by GC–MS analysis (Figs. S4 and S5). These results demonstrate that the reaction products were phenol, benzoate, and 2-PPE (i.e., TUS-SO1 converts 2-PAP to phenol, benzoate, and 2-PPE) (Fig. 4).

### Growth of TUS-SO1 on different carbon sources

**Figure 5:**
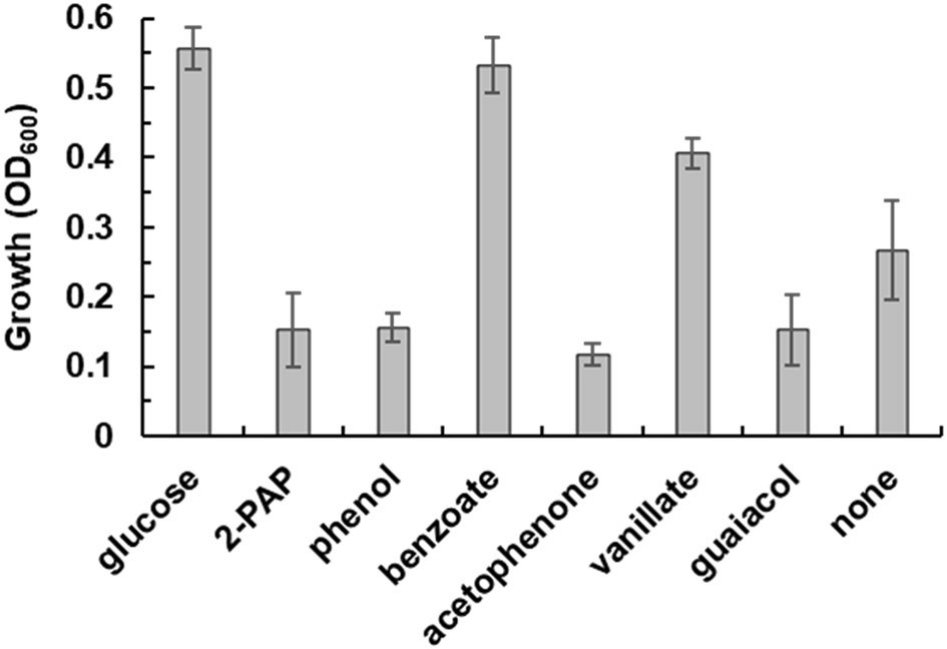
Growth of strain TUS-SO1 on different carbon sources. Strain TUS-SO1 was cultivated for 48 h in basal liquid medium supplemented with each indicated carbon source (1 mM) and yeast extract (0.5 g/L). The OD_600_ value at the time of inoculation was 0.03. Data are the average of five independent experiments, and error bars indicate the standard deviation from the mean.

We examined the growth of strain TUS-SO1 on different carbon sources. Strain TUS-SO1 was cultivated for 48 h in liquid basal medium supplemented with various organic compounds as a carbon source at a concentration of 1 mM. Yeast extract (0.5 g/L) was added to the medium as a cofactor, because this strain exhibited little or no growth on all carbon sources tested in the absence of yeast extract. Strain TUS-SO1 exhibited significant growth on glucose, benzoate, and vanillate (Fig. 5). In contrast, on medium supplemented with 2-PAP, phenol, acetophenone, or guaiacol, growth was lower as compared with that in the absence of these compounds (Fig. 5). These results indicate that strain TUS-SO1 grows weakly on medium containing 0.5 g/L yeast extract in the absence of the compounds, but its growth is inhibited in their presence. We also examined the growth of strain TUS-SO1 on medium containing 2-PAP (0.1 mM) in addition to glucose. As a consequence, the growth of strain TUS-SO1 on glucose was almost completely inhibited by 2-PAP (OD_600_, 0.04). Furthermore, HPLC analysis revealed that levels of benzoate and vanillate decreased after cultivation, whereas levels of the other aromatic compounds did not (Fig. S6). Overall, these data demonstrate that strain TUS-SO1 can assimilate glucose, benzoate, and vanillate but not 2-PAP, phenol, acetophenone, or guaiacol under the conditions examined in this experiment.

### Time course of 2-PAP transformation by TUS-SO1

We investigated in more detail the transformation of 2-PAP by strain TUS-SO1 when cultivated on carbon sources that the strain can assimilate (i.e., glucose, benzoate, and vanillate). TUS-SO1 cells were collected after cultivation for 48 h and then suspended in liquid basal medium supplemented with 0.1 g/L yeast extract and 1% (v/v) Tween 80 and incubated with 1 mM 2-PAP as a substrate for 72 h. Strain TUS-SO1 exhibited 2-PAP–transforming activity when cultivated on every carbon source tested, producing 0.9 mM, 0.6 mM, and 0.6 mM phenol from 1 mM 2-PAP when cultivated on glucose, benzoate, and vanillate, respectively. No 2-PAP–transforming activity was detected in the supernatants collected after cultivation.

**Figure 6:**
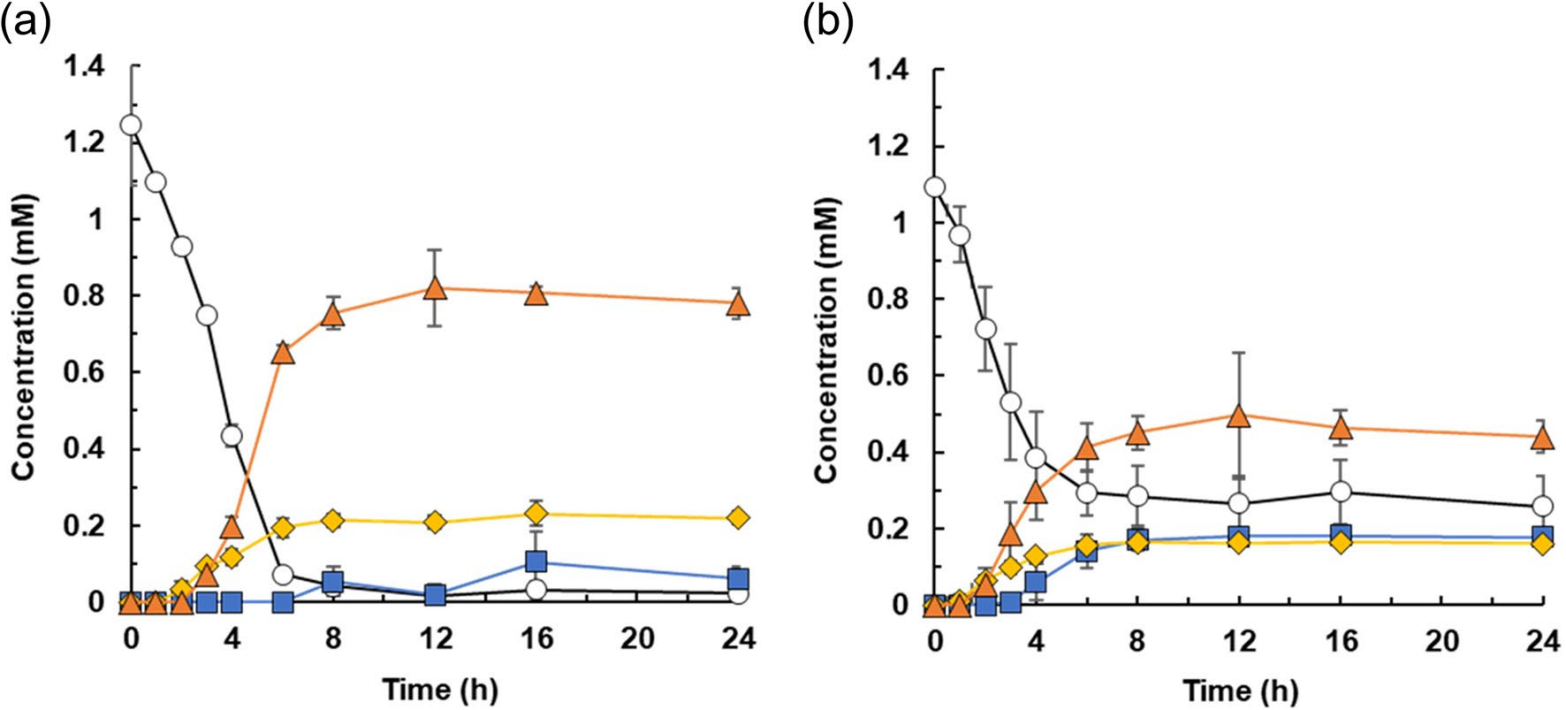
Time course of 2-PAP transformation by strain TUS-SO1. TUS-SO1 cells immediately after cultivation on glucose (**a**) or after pretreatment with 2-PAP for 4 h (**b**) were suspended in liquid basal medium supplemented with 0.1 g/L yeast extract and 1% (v/v) Tween 80. The cell suspension (OD_600_, 2.0) was incubated with 1 mM 2-PAP, and concentrations of 2-PAP (circles), phenol (triangles), benzoate (squares), and 2-PPE (diamonds) were determined at various time points. Data are the average of three independent experiments, and error bars indicate the standard deviation from the mean.

We also conducted a detailed examination of the transformation of 2-PAP by TUS-SO1 cells cultivated on glucose. The results of a time course analysis of 2-PAP transformation are shown in Fig. 6a. Glucose-grown TUS-SO1 cells converted 1 mM 2-PAP within 12 h. As the level of 2-PAP decreased, that of 2-PPE increased, reaching 0.2 mM in 12 h. Production of phenol began after 3 h, reaching 0.8 mM in 12 h. Benzoate was also produced, but this compound did not accumulate in the reaction mixture. These results demonstrate that strain TUS-SO1 converted 2-PAP to 2-PPE and generated phenol and benzoate as ether bond cleavage products (Fig. 4).

We next examined the effect of 2-PAP on the induction of 2-PAP-transforming activity of strain TUS-SO1. Glucose-grown TUS-SO1 cells were incubated with 2-PAP for 4 h (Fig. 6a) and then collected and further incubated with 1 mM 2-PAP (Fig. 6b). The lag time before production of phenol from 2-PAP (2 h, Fig. 6b) was shorter as compared with that of the glucose-grown cells (3 h, Fig. 6a). These results suggest that enzymes involved in the transformation of 2-PAP were partially induced by 2-PAP. After pretreatment with 2-PAP, TUS-SO1 cells were also incubated with 2-PPE, phenol, and benzoate. Strain TUS-SO1 did not transform 2-PPE or phenol but completely consumed benzoate. These results suggest that 2-PPE and phenol are the end products of the 2-PAP transformation pathway and that the low-level accumulation of benzoate in the reaction mixture is ascribed to the degradation of benzoate by strain TUS-SO1 (Fig. 4).

We further examined the transforming activity of strain TUS-SO1 toward another commercially available LMC that has a ketone group, MPHPV (Fig. S7), because this strain exhibited the activity toward the ketone compound 2-PAP but not the alcohol compound 2-PPE. After pretreatment with 2-PAP, TUS-SO1 cells were incubated with MPHPV. However, strain TUS-SO1 exhibited no activity toward MPHPV.

## Discussion

In this report, we describe the isolation of 2-PAP–transforming microorganisms and the detailed characterization of one strain, *Acinetobacter* sp. TUS-SO1. We first isolated microorganisms using medium supplemented with humic acid, which is a mixture of soil-derived organic compounds including aromatic polymers^23–26^. This medium is suitable for selecting a wide variety of microorganisms capable of transforming such compounds. The isolated microorganisms were subsequently subjected to a colorimetric assay for 2-PAP ether bond–cleaving activity (Fig. 1). This screening procedure resulted in the isolation of 8 strains of microorganisms, including 7 bacteria and 1 fungus (Fig. 3 and Table 1). To our knowledge, these are the first microorganisms that have been shown to cleave the ether bond of 2-PAP, although several microorganisms have been reported to cleave the ether bond of other LMCs such as guaiacylglycerol-β-guaiacyl ether^13,14^. The isolated bacteria belonged to 4 genera, *Acinetobacter*, *Streptomyces*, *Cupriavidus*, and *Nocardioides*. Several *Acinetobacter* and *Streptomyces* strains reportedly degrade lignin-related compounds such as guaiacylglycerol-β-guaiacyl ether and veratrylglycerol-β-guaiacyl ether^30–32^. Enzymes involved in the degradation of these compounds by *Acinetobacter* strains have not been identified, but degradation of lignin-related compounds by *Streptomyces* strains reportedly involves peroxidase and laccase^32,33^. A *Cupriavidus* strain was recently found to degrade kraft lignin^34^. To date, no reports have described the degradation of lignin-related compounds by *Nocardioides*.

*Acinetobacter* sp. TUS-SO1 assimilated glucose, benzoate, and vanillate but not 2-PAP, phenol, acetophenone, or guaiacol (Fig. 5). It is interesting to note that although strain TUS-SO1 cannot grow on 2-PAP, glucose-grown TUS-SO1 cells can convert 2-PAP to benzoate, which the strain is able to assimilate. This phenomenon was attributed to the 2-PAP–mediated inhibition of the growth of strain TUS-SO1 (Fig. 5). We also found that the growth of strain TUS-SO1 on glucose was almost completely inhibited by the addition of 0.1 mM 2-PAP to the medium. These results demonstrate the value of the screening procedure described above, because strain TUS-SO1 would not have been isolated using a screening method based on observation of growth on 2-PAP.

*Acinetobacter* sp. TUS-SO1 converted 2-PAP to phenol, benzoate, and 2-PPE (Figs. 2 and 6). Figure 4 shows the proposed pathway of 2-PAP transformation by strain TUS-SO1. Approximately 80% of 2-PAP was converted to phenol via ether bond cleavage, with the generation of benzoate (Fig. 6). We confirmed that benzoate was then degraded further. The residual 20% of 2-PAP was converted to 2-PPE via reduction of the carbonyl group. The generation of phenol and benzoate indicates that strain TUS-SO1 oxidatively cleaves the ether bond of 2-PAP (Fig. 4). The enzyme responsible for this reaction differs from reductive β-etherase, which has been well studied^4,5,10–12^, because β-etherase would convert 2-PAP to phenol and acetophenone if it exhibited catalytic activity toward 2-PAP. It is possible that oxidases such as peroxidase and laccase are involved in 2-PAP transformation by strain TUS-SO1. As these oxidases non-selectively degrade lignin-related compounds via a radical-based mechanism, a variety of products, which are generally difficult to quantify, are generated^32,33^. In contrast, strain TUS-SO1 selectively transformed 2-PAP to generate phenol and benzoate. Thus, an enzyme other than peroxidase and laccase might catalyze the reaction in strain TUS-SO1. In chemical transformation, it was reported that 2-PAP is converted to phenol and benzoic acid by Baeyer–Villiger oxidation (Fig. 4)^17^. These products of chemical transformation are same as those generated by strain TUS-SO1. Another possibility is that a type of Baeyer–Villiger monooxygenase catalyzes the ether bond cleavage by strain TUS-SO1 (Fig. 4). Strain TUS-SO1 exhibited no activity toward MPHPV. It is possible that the methoxy, hydroxy, and/or hydroxymethyl groups in MPHPV hamper the reaction of this molecule with the enzyme responsible for the 2-PAP transformation or the entrance of this molecule into to the cells (Fig. S7).

In conclusion, using a direct screening approach based on ether bond–cleaving activity, we isolated a variety of microorganisms that transform 2-PAP. The strategy used in this study should be generally applicable for screening microorganisms that cleave the ether bond of other lignin-related compounds. Furthermore, we demonstrated that one of the isolated microorganisms oxidatively and selectively cleaves the ether bond of 2-PAP. These microorganisms may play important roles in the degradation of lignin-related compounds in nature. In addition, the isolated organisms might be utilized for lignin valorization, especially the conversion of low-molecular-weight compounds that have chemical structures similar to 2-PAP. Further investigations will therefore focus on characterizing the enzymes involved in 2-PAP transformation, including examinations of their substrate specificities.

## Materials and methods

### Chemicals and cultivation media

Nitrohumic acid and 2-PAP were purchased from Tokyo Kasei (Tokyo, Japan). 2-PPE was purchased from Combi-Blocks (San Diego, CA, USA). MPHPV was purchased from Fujifilm Wako Chemicals (Osaka, Japan). Bacto yeast extract was purchased from Thermo Fisher Scientific (Waltham, MA, USA). Tween 80 was purchased from MP Biomedicals (Illkirch, France). All other chemicals were of analytical grade. Basal medium contained (per liter) KCl (1.71 g), Na_2_HPO_4_ (0.50 g), MgSO_4_·7H_2_O (0.05 g), CaCO_3_ (0.02 g), FeSO_4_·7H_2_O (0.01 g), and (NH_4_)_2_SO_4_ (1.50 g) (pH 7.2).

### Isolation of microorganisms

In the first screening, we isolated microorganisms that utilized nitrohumic acid as a carbon source for growth. Nitrohumic acid, which is a kind of humic acid, is produced from humic substances through oxidation with nitric acid, and have been used for cultivation of microorganisms^35^. Basal medium was supplemented with 1 g/L nitrohumic acid and 18 g/L Bacto agar, and 0.8% (w/v) sodium hydroxide aqueous solution (10 mL) was used to dissolve 1 g of nitrohumic acid. The pH value of the final medium containing nitrohumic acid was adjusted to 7.2. Each soil sample was suspended in saline, and the suspension was spread onto the solid basal medium. Microorganisms from soil samples were cultivated at 30 °C for 1 or 2 weeks. Colonies that appeared on the plate were then streaked onto fresh medium. Following cultivation on the solid basal medium, colonies that formed in the presence of nitrohumic acid were selected as candidate microorganisms capable of utilizing nitrohumic acid for growth^27^.

### Assay of 2-PAP ether bond cleavage

In the second screening, we assayed the isolated microorganisms for ability to cleave the ether bond of 2-PAP. Microbial cells cultivated on solid basal medium supplemented with nitrohumic acid were used for assays of 2-PAP ether bond cleavage. Cells were suspended in liquid basal medium supplemented with 0.1 g/L yeast extract and 1% (v/v) Tween 80. The reaction mixture (250 μL) contained cell suspension (OD_600_, 1–2), 2-PAP (5 mM for Gibbs assay, 1 mM for HPLC and GC–MS analysis), and dimethyl sulfoxide (DMSO, 1% [v/v]). The reactions were performed at 30 °C for 72 h with shaking. Strains producing phenol via cleavage of the ether bond of 2-PAP were selected using a colorimetric assay. Gibbs reagent (final concentration, 10 mM), which is sensitive to phenol, was added to the reaction mixture, and microorganisms that exhibited a color change were selected as positive strains^28,29^. Products generated by the microorganisms after 2-PAP transformation were also analyzed using HPLC and GC–MS, as described below.

### Taxonomic identification of microorganisms

The isolated bacteria and fungus strains were taxonomically identified based on sequencing of the 16S rRNA gene or ITS1 region, respectively. Bacterial DNA was amplified from colonies by polymerase chain reaction (PCR) using forward primer 5′-AGAGTTTGATCCTGGCTCAG-3′ and reverse primer 5′-AAGGAGGTGATCCAGCCGCA-3′^36,37^. Fungal DNA was amplified using forward primer 5′-GTAACAAGGT(T/C)TCCGT-3′ and reverse primer 5′-CGTTCTTCATCGATG-3′^38^. PCR was performed using KOD One PCR master mix (Toyobo, Osaka, Japan) according to the manufacturer’s recommendations under the following conditions: 35 cycles of 98 °C for 10 s, 58 °C for 5 s, and 68 °C for 8 s, followed by 25 °C for 2 min. After purification, the amplified DNAs were sequenced by Eurofins (Tokyo, Japan). The sequences were then compared against those in the GenBank database using BLASTN (https://blast.ncbi.nlm.nih.gov/Blast.cgi). MEGA software (https://www.megasoftware.net/) was used to align the sequences and construct a neighbor-joining phylogenetic tree^36,37^. The nucleotide sequences of the 16S rRNA gene and ITS1 region of the isolated microorganisms were submitted to GenBank under assigned accession numbers (Table 1).

### Cultivation of strain TUS-SO1

Strain TUS-SO1 was cultivated in liquid basal medium supplemented with various organic compounds as a carbon source at a concentration of 1 mM (see Fig. 5). DMSO (1% [v/v]) was added to the medium to dissolve aromatic compounds. Yeast extract (0.5 g/L) was added to the medium as a cofactor. Strain TUS-SO1 was cultivated at 30 °C for 48 h with reciprocal shaking in test tubes containing 2 mL of medium. The OD_600_ value at the time of inoculation was 0.03. Bacterial growth was determined by measuring the OD_600_.

### Reactions using strain TUS-SO1

TUS-SO1 cells cultivated in liquid basal medium supplemented with organic compounds were harvested by centrifugation and suspended in liquid basal medium supplemented with 0.1 g/L yeast extract and 1% (v/v) Tween 80. The reaction mixture (250 μL) contained cell suspension (OD_600_, 2.0), 2-PAP (1 mM), and DMSO (1% [v/v]). The reactions were performed at 30 °C with shaking.

### Product analysis

Reaction products were detected by HPLC analysis using an LC-20 system (Shimadzu, Kyoto, Japan) equipped with a Cosmosil 5C18-PAQ packed column (4.6 × 250 mm, Nacalai Tesque, Kyoto, Japan)^39,40^. The post-reaction mixture was acidified by the addition of HCl (pH 2–3), and methanol (500 μL) was then added. The solution was vigorously shaken and centrifuged, and the resulting supernatant (10 μL) was injected into the HPLC system. Mobile phases were water with 0.1% formic acid (A) and methanol (B). A gradient of mobile phase B was programmed as follows: start ratio of 20%, hold at 20% for 13 min, increase to 50% for 1 min, increase linearly to 80% for 10 min, increase to 100% for 1 min, hold at 100% for 6 min, decrease to 20% for 1 min, and hold at 20% for 8 min. The flow rate was 0.5 mL/min. Compounds were detected spectrophotometrically at a wavelength of 200 nm. The amounts of 2-PAP, phenol, benzoate, and 2-PPE were calculated from standard calibration curves that were made using the commercially available compounds. UV–visible absorption spectra were acquired using a photodiode array detector (SPD-M20A, Shimadzu).

GC–MS analysis was performed using a 6890 system (Agilent, Palo Alto, CA, USA) equipped with a DB-5 ms column (0.32 × 30 mm, Agilent)^41,42^. The post-reaction mixture was acidified by the addition of HCl (pH 2–3), and ethyl acetate (1 mL) was then added. The solution was vigorously shaken and centrifuged, and the resulting supernatant (1 μL) was injected into the GC–MS system. For analysis of benzoic acid, the extracts were treated with BSTFA + TMCS (Supelco, Bellefonte, PA, USA) to substitute a trimethylsilyl group for a hydrogen in the carboxyl group. The carrier gas was helium, and the flow rate was 2.0 mL/min. The injection and detection temperatures were maintained at 250 °C and 280 °C, respectively. The oven temperature was programmed as follows: start temperature of 60 °C, hold for 1 min at start temperature of 60 °C, increase to 250 °C at a rate of 10 °C/ min, and hold for 5 min at finish temperature of 250 °C.

## Supplementary Information

Supplementary Information.
