# Supplementary material for: Isolation and characterization of microorganisms capable of cleaving the ether bond of 2-phenoxyacetophenone
Source: Sci Rep. 2022 Feb 21;12:2874. doi: 10.1038/s41598-022-06816-1 (PMC8861056; doi:10.1038/s41598-022-06816-1)
Supplement: Supplementary file 1 — Supplementary Information. [file 41598_2022_6816_MOESM1_ESM.pdf]

**Supplementary Information**

# Isolation and characterization of microorganisms capable of cleaving the ether bond of 2-phenoxyacetophenone

Saki Oya[1], Satoshi Tonegawa[1], Hirari Nakagawa[1], Hiroshi Habe[2], Toshiki Furuya[1]

---

[1]Department of Applied Biological Science, Faculty of Science and Technology, Tokyo University of Science, 2641, Yamazaki, Noda, Chiba 278-8510, Japan. [2]Environmental Management Research Institute, National Institute of Advanced Industrial Science and Technology (AIST), 16-1 Onogawa, Tsukuba, Ibaraki 305-8569, Japan.

Correspondence and requests for materials should be addressed to T.F. (email: tfuruya@rs.tus.ac.jp)

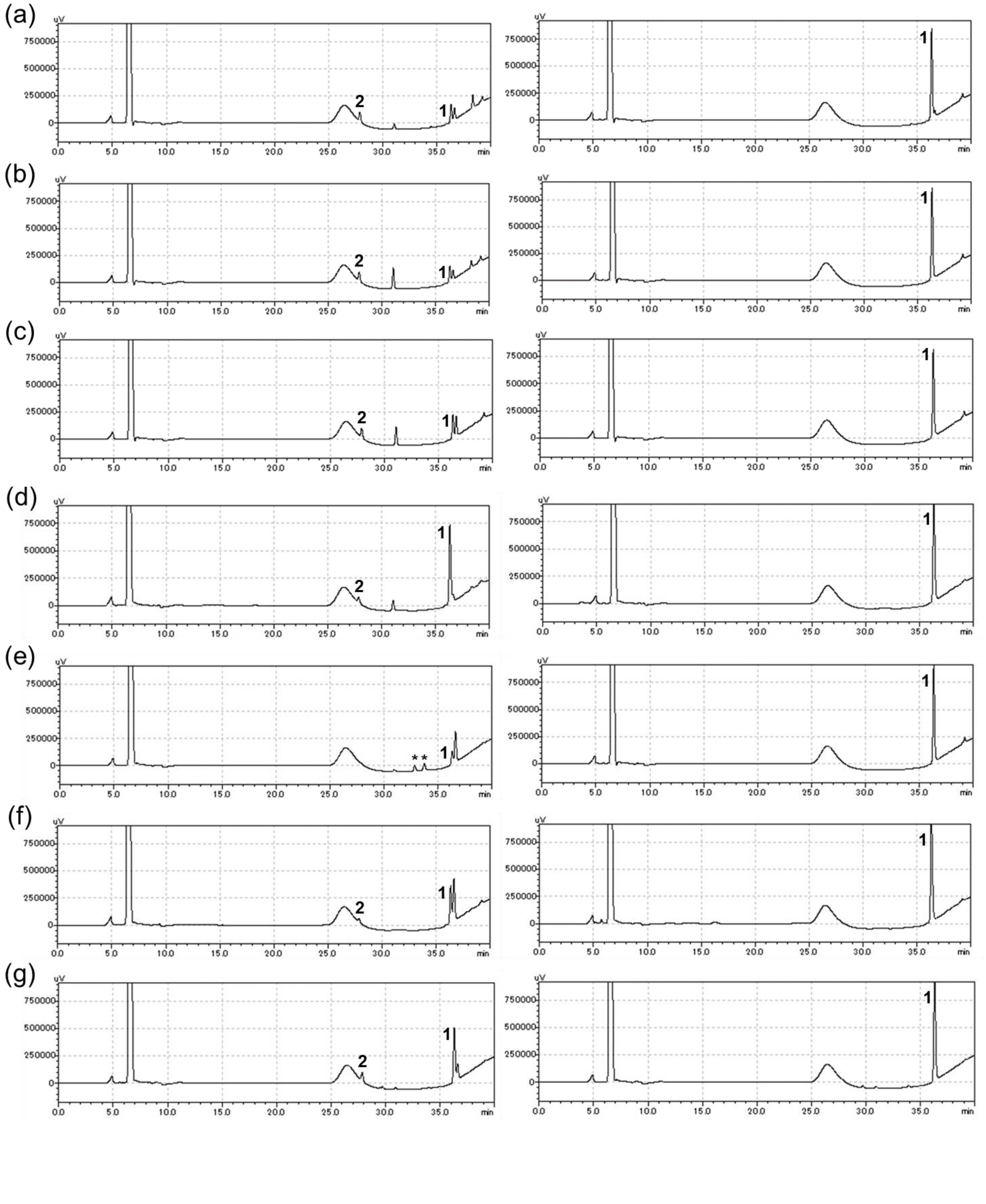
(a)
(b)
(c)
(d)
(e)
(f)
(g)
uV
min

**Figure S1.** HPLC analysis of the reaction products formed by incubation of the isolated strains other than TUS-SO1 with 2-PAP. Microbial cells after cultivation on solid basal medium supplemented with nitrohumic acid were incubated with 1 mM 2-PAP for 72 h. Cells of strain TUS-SO2 (a), TUS-SO3 (b), TUS-SO5 (c), TUS-SO6 (d), TUS-SO7 (e), TUS-SO8 (f), or TUS-SO9 (g) were incubated with 2-PAP. The reaction was performed using intact cells (left chromatograms) or autoclaved cells (right chromatograms). Peaks 1 (at 36.3 min), and 2 (at 27.7 min) correspond to 2-PAP and phenol, respectively. In (e), peaks shown by an asterisk (at 32.9 min and 33.8 min) were detected instead of phenol.

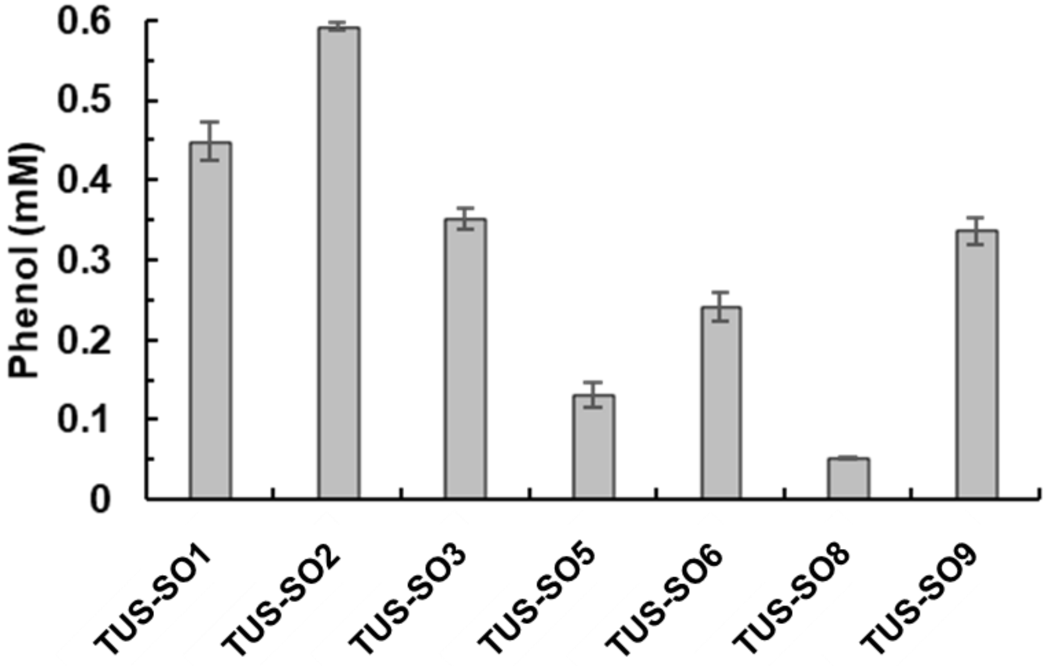


**Figure S2.** 2-PAP ether bond–cleaving activity of the isolated microorganisms. Microbial cells after cultivation on solid basal medium supplemented with nitrohumic acid were suspended in liquid basal medium supplemented with 0.1 g/L yeast extract and 1% (v/v) Tween 80. The cell suspension ($OD_{600}$, 2.0) was incubated with 1 mM 2-PAP for 72 h. The amount of phenol was determined using HPLC. Data are the average of three independent experiments, and error bars indicate the standard deviation from the mean.

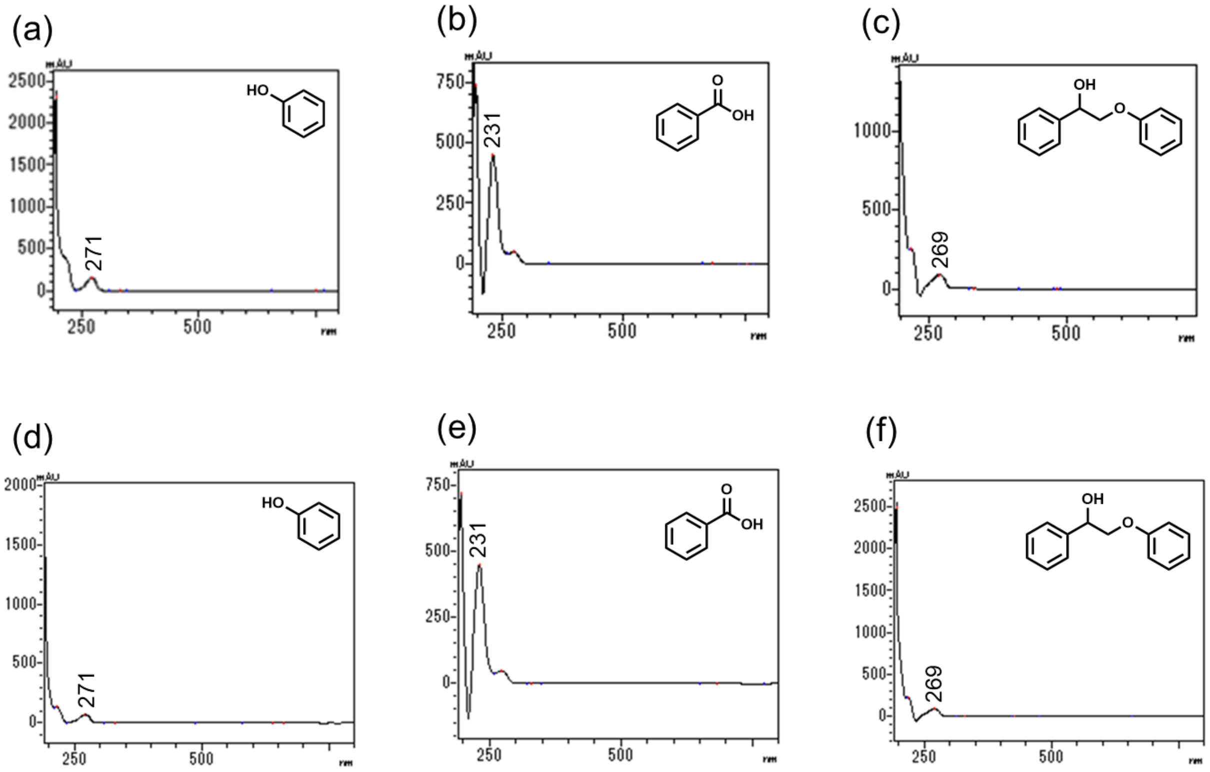


**Figure S3.** UV-visible absorption analysis of the reaction products formed from 2-PAP by strain TUS-SO1. The UV-visible absorption spectra for peak 2, 3, and 4 in Figure 2 are shown in (a), (b), and (c), respectively. These spectra were consistent with those of authentic samples of phenol (d), benzoate (e), and 2-PPE (f), respectively.

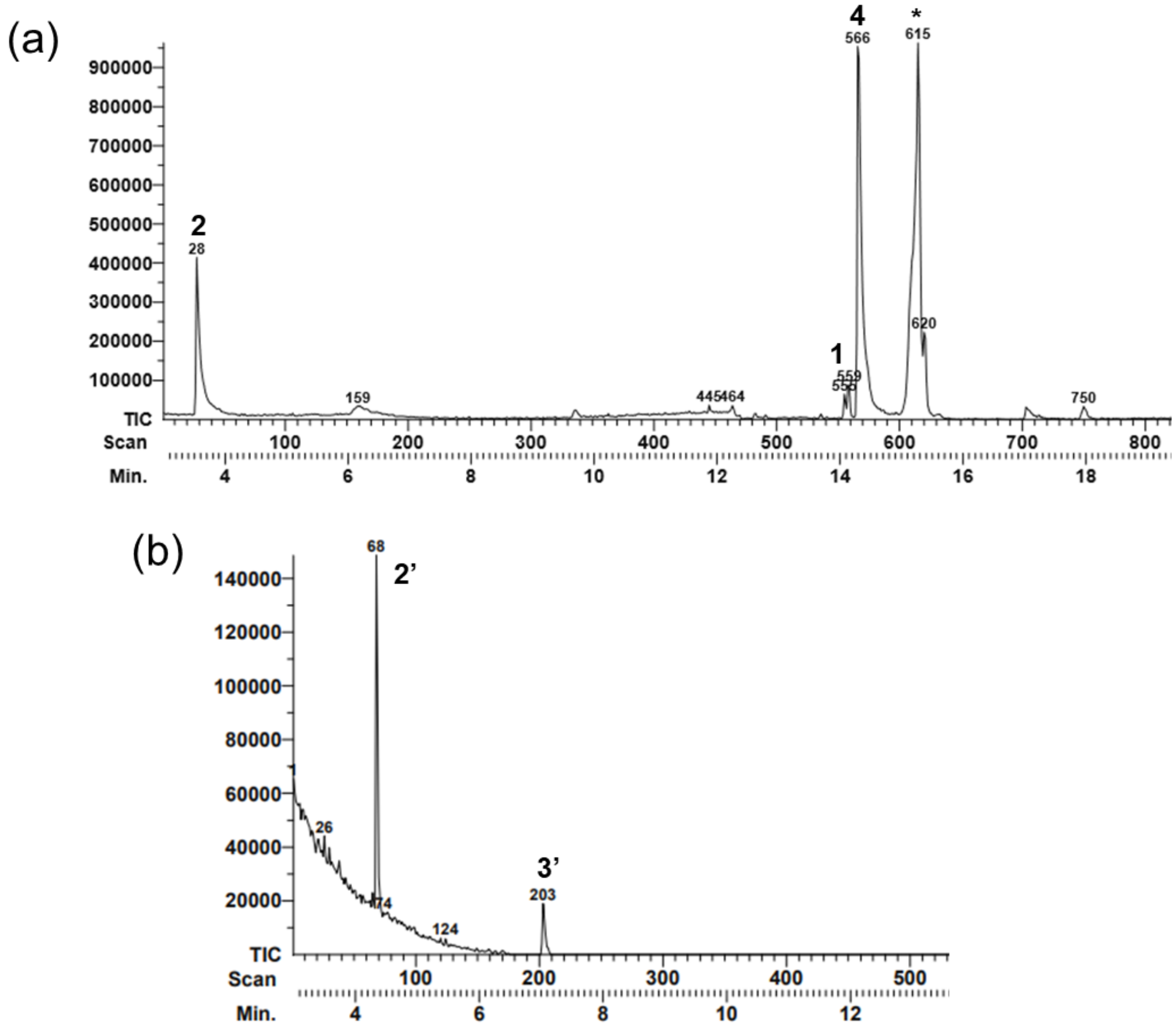


**Figure S4.** GC analysis of the reaction products formed by incubation of strain TUS-SO1 with 2-PAP. Microbial cells after cultivation on solid basal medium supplemented with nitrohumic acid were incubated with 1 mM 2-PAP for 72 h. In (a), Peaks 1 (at 14.1 min), 2 (at 3.5 min), and 4 (at 14.3 min) correspond to 2-PAP, phenol, and 2-PPE, respectively. The compound corresponding to a peak shown by an asterisk (at 15.3 min) could not be identified in this study. In (b), the reaction mixture was treated with trimethylsilyl derivatization reagent. Peaks 2' (at 4.3 min) and 3' (at 7.0 min) correspond to the derivatives of phenol and benzoate, respectively.

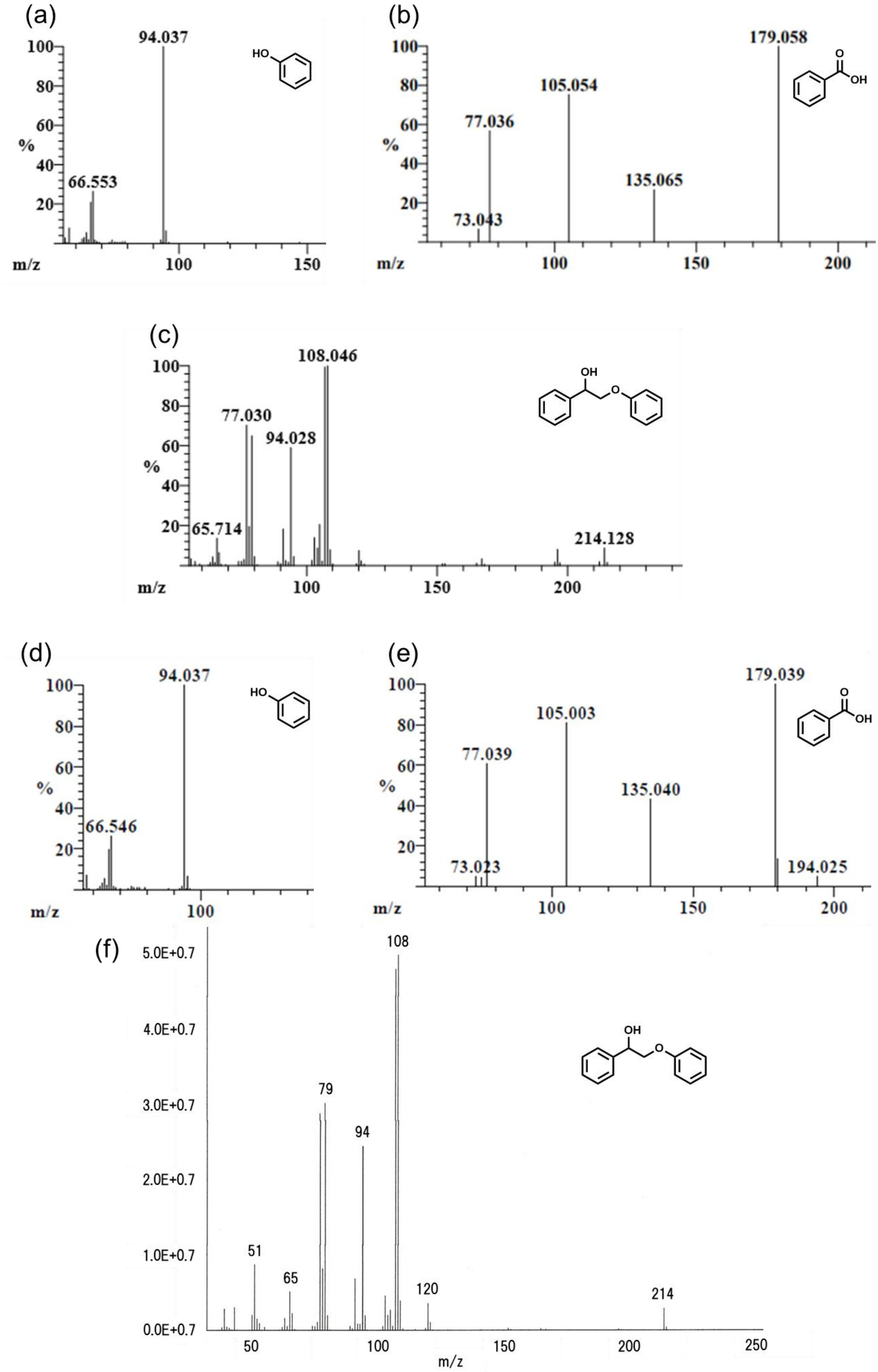

(a)
94.037
66.553
HO
%
m/z
(b)
179.058
105.054
77.036
135.065
73.043
OH
%
m/z
(c)
108.046
77.030
94.028
65.714
214.128
OH
O
%
m/z
(d)
94.037
66.546
HO
%
m/z
(e)
179.039
105.003
77.039
135.040
73.023
194.025
OH
%
m/z
(f)
108
79
94
51
65
120
214
OH
O
m/z

**Figure S5.** GC-MS analysis of the reaction products formed from 2-PAP by strain TUS-SO1. GC-MS spectra corresponding to peak 2, 3’, and 4 in Figure S4 are shown in (a), (b), and (c), respectively. When benzoic acid was analyzed in (b), a hydrogen in the carboxyl group was substituted with a trimethylsilyl group to detect this compound. These spectra were consistent with those of authentic samples of phenol (d), trimethylsilylated benzoate (e), and 2-PPE (f), respectively. The spectrum in (f) was determined by GFC Instrumental Analysis Service (Hokkaido, Japan).

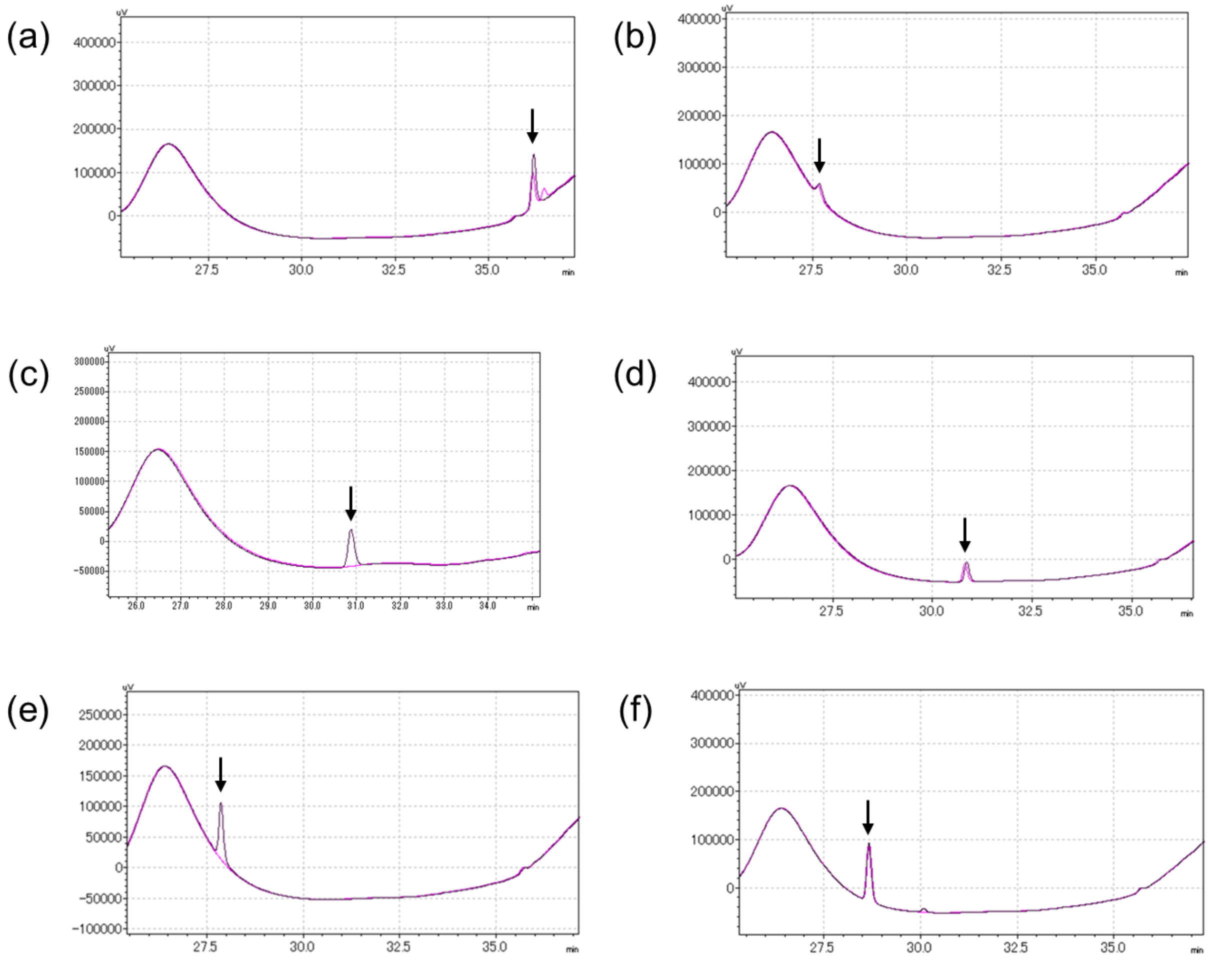


**Figure S6.** HPLC analysis of the carbon sources for growth of strain TUS-SO1. Strain TUS-SO1 was cultivated for 48 h in liquid basal medium supplemented with 2-PAP (a), phenol (b), benzoate (c), acetophenone (d), vanillate (e), or guaiacol (f). The incubation was performed with inoculation of cells (pink chromatograms) or without inoculation of cells (black chromatograms). Peaks corresponding the carbon sources are shown by arrows. In (c) and (e), peaks shown by arrows decreased, when the incubation was performed with inoculation of cells.

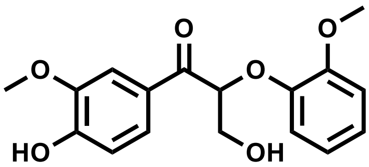

**Figure S7.** Molecular structure of MPHPV.
